# scientific, social, ecological rationality and public attitudes toward genetically modified food in China: based on the survey of urban residents in three cities of China

**DOI:** 10.1080/21645698.2026.2728190

**Published:** 2026-09-09

**Authors:** Hui Li, Zhenjing Pang, Tian Yu

**Affiliations:** a Chengdu Future Intelligent Manufacturing Industry Development Research Center, Chengdu Vocational & Technical College of Industry, Chengdu, China; b School of Public Administration, Sichuan University, Chengdu, China

**Keywords:** Ecological rationality, GM food, institutional trust, public attitudes, scientific rationality, social rationality

## Abstract

Genetically modified (GM) technology is crucial for global food security, but its acceptance is often constrained by public attitudes. This study investigates how three types of rationality (scientific, social, and ecological) are associated with attitudes toward GM food in China, and examines the mediating role of institutional trust. Based on questionnaire data from 1020 Chinese urban residents, structural equation modeling showed that scientific rationality was positively associated with perceived benefits but not with perceived risks. Social rationality was negatively associated with perceived benefits and positively with perceived risks. Ecological rationality was positively associated with perceived risks only. Institutional trust directly predicted public attitudes and mediated the relationships between rationality types and perceived benefits/risks. The indirect path from scientific rationality via trust and perceived risks was not significant, suggesting this group’s positive attitudes stem from benefit-focused processing. The model explained 53.6% of attitude variance. Findings demonstrate that public attitudes are rooted in value orientations and channeled through institutional trust, offering implications for targeted communication, value dialogue, and trust‑building in food risk governance.

## 1. Introduction

Agricultural genetically modified (GM) technology is recognized as one of the key approaches to addressing global food security challenges, increasing agricultural productivity, and meeting public diverse preferences.^1^ However, due to its uncertainty, GM technology still faces complex public controversy and acceptance challenges globally.^2,3^ In particular, this kind of genetically modified technology, which uses biotechnological methods to introduce specific exogenous genes (usually from different species) into the genome of the target organism, enabling it to acquire new and stably inheritable traits (such as resistance to pests, tolerance to herbicides, etc.). This controversy is particularly evident in China.^4^ Discussions around GM food have extended beyond the purely scientific realm to multiple dimensions, including social, cultural, and ethical spheres,^5^ prompting a reexamination of the relationship between science, society, and nature in the context of reflexive modernization. In the early 2010s, there was an extremely intense debate on the risks of genetically modified foods on the Chinese Internet, almost turning into a “trust battle” that divided the society. This debate reached its first climax around 2010, with the core trigger being the Ministry of Agriculture’s issuance of safety certificates for two types of genetically modified rice at the end of 2009. This was regarded by the public as the prelude to the commercialization of genetically modified staple foods, instantly igniting public anxiety. On the internet, supporters and opponents formed two opposing camps represented by public figures such as Fang Zhouzi and Cui Yongyuan. The focus of the debate quickly expanded from pure food safety to concerns about food sovereignty, economic security, and even national survival. During this period, various highly impactful rumors spread virally on forums and social media, with the authenticity of this information being difficult to determine, but they greatly fueled public panic. At the same time, the official and scientific responses were considered slow and ineffective, further highlighting the profound divisions within the scientific community and among the social elite. This debate has long transcended a simple scientific discussion and has evolved into a major social debate about food safety, government credibility, commercial interests, and technological ethics. This social debate profoundly reflects the rationality and value split regarding genetically modified technology. Due to expertise barriers, information asymmetry, and cognitive limitations, the public tends to rely on subjective values shaped by personal experiences, beliefs, and trust when assessing the risks of GM food, while public attitudes and risk perception pathways based on objective cognitive systems are often ineffective.^6,7^ That is, subjective value systems often play a stabilizing and critical role in shaping attitudes and risk perceptions toward GM food.^8,9^

The risk of GM food is a subjectively perceived risk whose objective manifestation is not yet apparent.^10^ Therefore, the controversy is not only reflected in the differences in public perceptions of risks and benefits, but also stems from differences in the subjective value systems used to understand GM food, a phenomenon known as “framing.”^11^ The subjective value system can be conceptualized as scientific rationality, social rationality, and ecological rationality. These three types of rationality differ fundamentally in the definition of the nature of risk, judgment of controllability, and value orientation, and influence public attitudes and risk perception of GM food through different forms, dimensions, and intensities.^12,13^ Although studies have examined the effects of various isolated factors such as rationality, risk perception, or trust on attitudes separately,^14–16^ there is still limited understanding of the pathways by which pluralistic rationalities interact with institutional trust and jointly predict public attitudes. Particularly in China, institutional trust has been shown to be a key variable influencing public attitudes toward emerging technologies.^17^ The relationship between the public and science is no longer one of mere subordination, but a comprehensive value-judging mechanism intertwined with trust and rationality, resulting in very different risk/benefit perception and public attitudes toward GM food.

To provide new research evidence, this study argues that it is necessary to gain a deeper understanding of the subjective value system and to further examine how scientific rationality, social rationality, and ecological rationality, as the deep value structures shaping belief systems, interact with institutional trust to influence perceived benefits and risks and ultimately predict the Chinese public’s attitudes toward GM food. Based on the questionnaire data, we empirically tested the proposed theoretical model using Structural Equation Modeling. The findings not only provide a new theoretical framework for in-depth analysis of public attitude formation pathways but also lay a solid empirical foundation for the development of more targeted and effective risk communication and policy intervention strategies.

## 2. Literature Review and Research Hypotheses

The Multi-Attribute Model (MAM) is a crucial framework for explaining people’s attitudes toward emerging technologies. It views attitudes as a weighted function of beliefs about product attributes: individuals first form subjective judgments (i.e., beliefs) about each attribute of the product, then integrate these beliefs by weighting them according to the importance of each attribute, and finally form an overall attitude.^18^ This model assumes a premise: individuals make judgments based on sufficient information, by carefully examining, comparing, and correlating various pieces of evidence. In other words, individuals usually require higher-quality objective information and knowledge in order to make attitude decisions. However, this premise is often difficult to meet in the context of GM food. When confronted with complex and uncertain issues such as GM food, individuals may be more inclined to make stable judgments by relying on their subjective values and heuristics rather than by seeking information and knowledge to complete a systematic analysis.^19,20^ Multiple studies have also found that there is a weak association between objective knowledge and public attitudes toward GM food.^14,21,22^ The objective cognitive system is not the core process by which the public forms attitudes; its influence is far less than that of the subjective value system.^23,24^

The Theory of Planned Behavior (TPB) provides an important theoretical framework for understanding how subjective value systems influence public attitudes.^25,26^ It holds that attitudes are associated with behavioral beliefs and the evaluation of behavioral outcomes. However, TPB does not explain where these beliefs come from. Instead, it treats them as given and does not further trace their origins.^9,27^ This study argues that scientific rationality, social rationality, and ecological rationality are precisely the underlying value structures that shape these beliefs. As the cognitive background for individuals to interpret external information, weigh the consequences of actions, and perceive social pressures, these three types of rationality predispose individuals to form different types of beliefs about GM food: scientific rationality generates beliefs concerning technological benefits and controllability; social rationality generates beliefs focusing on power distribution and procedural justice; ecological rationality generates beliefs regarding natural boundaries and ethical morality.^11–13^ These rationality-driven beliefs constitute the specific content of behavioral beliefs within the TPB framework.

These rationality-driven beliefs, in turn, manifest as the public’s perceptions of the risks and benefits of GM food. The Risk-benefit Analysis Framework provides a direct theoretical tool for understanding this transformation.^28^ This framework regards perceived risks and perceived benefits as direct predictors of attitudes, and has been confirmed by numerous studies on GM food.^10,29^ Furthermore, scholars have also focused on the crucial role of institutional trust in risk perceptions and attitudes toward GM technologies.^30,31^ Institutional trust serves as a heuristic cue for the public to simplify decision-making when information is incomplete, and may also directly shape attitude formation in specific cultural contexts.

**Figure 1.**
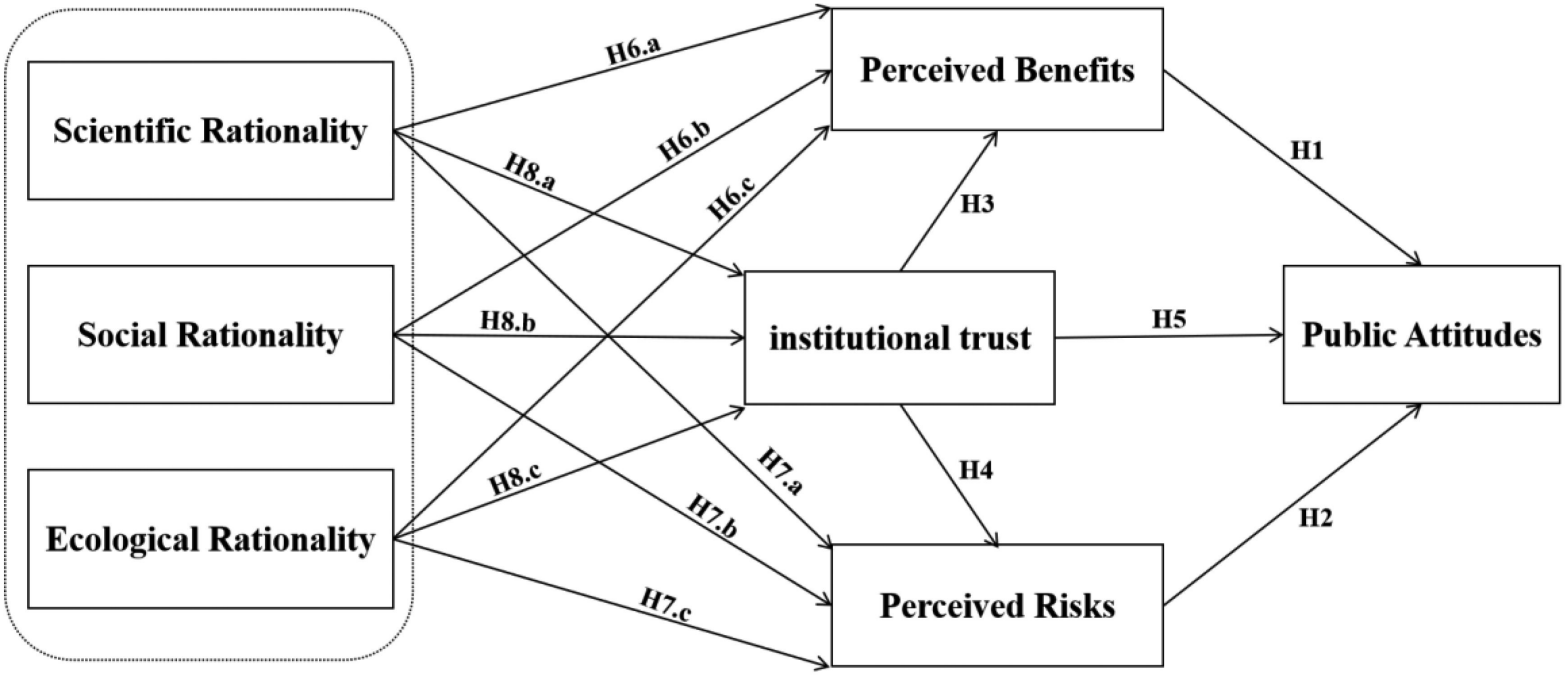
Theoretical model.

By integrating the above theories, this study constructed a conceptual model as shown in Figure 1. This model positions scientific, social, and ecological rationality as the initial value orientations that influence public perceptions of GM food. It examines how these three types of rationality, through the mediating role of institutional trust, affect public perceptions of risks and benefits, and ultimately predict public attitudes toward GM food. The proposed conceptualization and hypotheses are discussed below.

### 2.1. The Relationship Between Perceived Benefits/Risks and Public Attitudes

The Risk-benefit Analysis Framework provides a central explanatory mechanism for understanding public attitudes toward emerging technologies.^32,33^ The framework argues that an individual’s overall attitude toward a technology fundamentally stems from the perception of its potential benefits and risks. Regardless of objective facts, individuals are more likely to develop positive attitudes when they subjectively perceive that the benefits of a new technology outweigh its potential risks; conversely, negative attitudes emerge when risks are perceived to outweigh benefits.^34–36^

This Risk-benefit Analysis Framework has also received broad and consistent empirical support in studies of GM food.^29,37^ Specifically, public attitudes toward GM food are significantly and negatively influenced by their perceived risks, such as health and the environment, and positively influenced by their perceived benefits, such as price and taste of a given product.^38^ Notably, this analytical framework has been shown to be a stable proximal pathway for shaping public attitudes across cultures.^1,39^

In the comprehensive model constructed in this study, perceived benefits and perceived risks are positioned as proximal determinants that directly influence public attitudes. They are the direct basis for the public to make judgments, and they also form a key communication pathway connecting the deeper antecedent variables (rationality framework and institutional trust) to the final attitudes. Based on this solid theoretical and empirical foundation, we propose the following hypotheses:

H1: Perceived benefits positively predict public attitudes toward GM food.

H2: Perceived risks negatively predict public attitudes toward GM food.

### 2.2. The Relationship Between Institutional Trust and Perceived Benefits/risks

Institutional trust refers broadly to public confidence in the institutions responsible for governing emerging technologies, including government agencies, regulatory bodies, and scientific institutions.^40,41^ From the perspective of the intersection of social system theory and cognitive psychology, the dominant influence of institutional trust on the perception of risks and benefits of emerging technologies essentially stems from an adaptive simplification mechanism formed by humans’ invocation of the “cognitive miser” instinct in a highly complex risk society. According to Luhmann’s theory of system trust and the Heuristic-Systemic Information Processing Model (HSM), when the public confronts highly specialized technical objects with extremely high barriers, such as genetically modified technologies, they do not actively weigh their scientific mechanisms. Instead, they take “whether to trust the regulatory system and the scientific community” as a single core decision heuristic. Through this trust cue, they directly reduce technological uncertainty and thereby substantially replace the original knowledge cognitive path that required deep scientific literacy. When the procedural justice, regulatory transparency, or historical credit on which institutional trust relies are eroded by accidental events, the knowledge path that has been marginalized for a long time is difficult to provide alternative cognitive support because it cannot be immediately awakened. This reveals a fundamental law in modern technology governance: Institutional trust is not only a necessary compensation for limited human cognitive rationality and an inevitable choice for social operational efficiency, but also, due to its long-term suppression and substitution of knowledge paths, it becomes the most crucial stability fulcrum in the entire risk perception system and the most potential source of vulnerability. In the context of GM food, the most directly relevant institutions are the regulatory authorities that oversee risk assessment, approval, and monitoring. Therefore, this study focuses specifically on trust in these regulatory bodies as a core social asset in risk governance systems. In the case of GM food, institutional trust influences public attitudes through two main pathways.

On the one hand, institutional trust has an indirect impact by influencing perceptions of benefits and risks. When individuals lack specialized knowledge, trust in authoritative institutions is a key cue for simplifying complex decisions.^42^ A high level of institutional trust is a mechanism of “social reassurance” that reduces anxiety about unknown dangers, thereby reducing perceived risks. At the same time, it increases confidence in the promised benefits of the technology, thereby increasing the perceived benefits.^43,44^ A large body of empirical evidence confirms that institutional trust is a key antecedent variable in the public’s evaluation of the GM food risks and benefits.^45,46^

On the other hand, in high-context cultures like China, institutional trust may also have a direct normative impact on public attitudes. High-context cultures emphasize nonverbal communication, social hierarchy, and collective identity, and trust is often based on compliance with authority rather than personal calculation.^47,48^ Thus, as an expression of social conformity or groupthink, public attitudes may align with the official position, a process that is somewhat independent of individualized risk-benefit analysis frameworks.^31^ This direct “trust spillover” effect makes institutional trust a strong predictor of public attitudes.^17^

Based on the theoretical premise that institutional trust influences public attitudes through both indirect (via risk/benefit perceptions) and direct (normative influence) pathways, we propose the following hypotheses:

H3: Institutional trust positively predicts the perceived benefits of GM food.

H4: Institutional trust negatively predicts the perceived risks of GM food.

H5: Institutional trust positively predicts public attitude toward GM food.

### 2.3. The Relationship Between Rationality and Perceived Benefits/risks

The Cultural Theory of Risk views manufactured risks as constructs of specific values and belief systems, emphasizing their close connection to individual values and worldviews.^49,50^ The controversy over public perceptions of GM food does not stem solely from contradictory risk governance strategies, but rather from differences in subjective value systems for understanding GM food, which can be considered a rationality framing issue – that is, how the issue is framed by different rationality types.^51^ It is important to clarify that these types of rationality are conceptualized as analytical constructs that capture the core logic of each value orientation, rather than mutually exclusive empirical categories. In reality, individuals may hold mixed or context-dependent views, and the boundaries between the rationality frameworks can be fluid. However, as analytical tools, they provide a means for unraveling the different cognitive logics underlying the public debate regarding GM food. The study initially proposed a framework for analyzing the risk of GM food from the perspective of social rationality and scientific rationality, explaining the differences in the shape, dimension, and intensity of risk perception and public attitudes among people with different rationality value orientations.^11^ Specifically, the differences between scientific rationality and social rationality begin with a fundamental divergence in understanding GM food risks/benefits, which explains why there are differences in the types of hazard exposure, risk assessment, risk management, and risk communication among people in different risk perception structures.

Scientific rationality is the cognitive orientation that the public adopts when dealing with emerging technologies. It relies on scientific methods and logical thinking, using objective facts and quantifiable risks as the cognitive basis, to make a cautious judgment on the safety and effectiveness of the technology. It requires the public to go beyond intuition, emotions or fragmented information, adhere to the spirit of evidence-based reasoning, understand technological risks as probabilistic events, and weigh the pros and cons based on the existing scientific evidence. Scientific rationality insists on the use of instrumentalist and evidence-based approaches to perceive risk, attempting to define the risk of GM food as the probability of suffering harm or loss, implying that the goal of risk governance is to develop regulatory policies that maximize technological advances while meeting specific safety standards.^52^ Scientific rationality does not recognize the existence of unknown, unlimited risks and believes that existing technology is unable to detect such risks.^53^ Based on a demand orientation, scientific rationality attempts to construct a discourse of technological optimism by describing the advantages of genetically modified food.^54^ In addition, scientific rationality uses the principle of substantial equivalence and the anticipatory approach as risk assessment tools, requiring adequate scientific data on hazards when restricting the commercialization of GM food, and emphasizing that GM food should be presumed safe until objective risks are proven.^55,56^ Thus, under the scientific rationality perspective, the benefits of unrestricted release of GM food are given significant weight, while the uncertainty of harm is underestimated.

In contrast, social rationality extends the scrutiny of GM technology from the laboratory to the wider social sphere, arguing that the risks and benefits of the technology are profoundly influenced by power structures, economic interests, and cultural contexts.^11,57^ Social rationality is a cognitive dimension through which the public, from the perspective of the overall interests and fairness and justice of society, systematically considers the social structural changes, distribution patterns of interests, and interpersonal ethical relationships triggered by emerging technologies, based on the values of social harmony, inclusiveness, and public well-being. It requires the public to view technology within the complex social operation network and examine it not only from the average performance of technological effectiveness, but also from the differentiated impacts it has on different social strata, regions, and generations. In particular, it is vigilant against the unfair phenomenon where the technological dividends are monopolized by a few groups while the risks and costs are borne by the disadvantaged groups. Thus, distributive justice is regarded as an important criterion for evaluating technological development. Social rationality emphasizes the potential threat of GM food to society and attempts to construct a techno-skeptical discourse.^58,59^ The precautionary principle is the cornerstone of social rationality, and scientific uncertainty should not be a reason to postpone protective measures against unpredictable hazards.^60,61^ Risk assessment requires not only the use of scientific data, but also the consideration of some nonscientific perceptions.^62^ With the rise of knowledge communities, experts (scientific or policy) are no longer automatically regarded as the most credible source of knowledge for assessing the risks of GM food.^63^ Social rationality provides a framework for individuals to interpret the topic of GM food and construct meaning. As a toolkit, social rationality influences public attitudes toward GM food by linking technology to specific symbols, norms, and values through risk-semantic relations.

While scientific rationality and social rationality provide largely divergent interpretations of perceptions and attitudes toward GM food, a third type of rationality – ecological rationality – provides a fresh perspective for understanding risk and uncertainty associated with GM food. Ecological rationality is a thinking framework that the public adopts from the holistic perspective of harmonious coexistence between humans and nature. It examines emerging technologies within the broad context of the life community and the ecosystem, using long-term, systematic, and intergenerational equity as its value coordinates. It does not rely solely on anthropocentrism as a reference point. It emphasizes that the application of technology must follow the laws of nature’s limits and is vigilant against any technological impulses that may disrupt ecological balance, ecological ethics, or erode biodiversity. In the public’s attitude and perception, ecological rationality often manifests as a deep preference for the “naturalness” and “sustainability” of technology. Ecological rationality does not negate scientific rationality; rather, it reflects on and checks its instrumental tendencies. While the divergence between scientific rationality and social rationality reflects the blurred boundaries of technological embeddedness in society, ecological rationality focuses on the relationship between humans and nature.^64,65^ Characterized by non-negotiability and absolutism, ecological rationality illustrates how ethical principles of naturalness influence technological risk perception and attitude formation.^66,67^ Influenced by religious and traditional concepts, ecological rationality strongly opposes technological rationality that transcends natural law and order, and demonstrates a strong moral critique and ethical concern about unforeseen cross-generational environmental consequences in the short term.^20,68^ Ecological rationality attempts to move away from anthropocentrism by emphasizing that organisms deserve a special moral status. Ecological rationality advocates argue that GM food is morally unacceptable because it disrupts natural species boundaries.^69^

In sum, the rationality framework systematically guides individuals’ cognitive focus and judgmental criteria. While we acknowledge that these rationalities may overlap or interact in practice, treating them as ideal types allows us to isolate their distinctive cognitive logics. Therefore, we argue that they differentially influence the perceived benefits and risks of GM food and propose the following hypothesis:

H6: Different types of rationality predict the perceived benefits of GM food.

H6.a: Scientific rationality positively predicts perceived benefits.

H6.b: Social rationality negatively predicts perceived benefits.

H6.c: Ecological rationality negatively predicts perceived benefits.

H7: Different types of rationality predict the perceived risks of GM food.

H7.a: Scientific rationality negatively predicts perceived risks.

H7.b: Social rationality positively predicts perceived risks.

H7.c: Ecological rationality positively predicts perceived risks.

### 2.4. The Relationship Between Rationality and Institutional Trust

In risk societies, the public’s relationship with science has shifted from a deficit model to a reflexive or democratic model,^70,71^ which has made the public’s interactions with the government less about obedience based on authority, and more about trust based on subjective value judgments within the domain of scientific cognition. Institutional trust often depends on whether the public perceives the decision-makers in power as conforming to their value expectations.^72,73^ Public trust in official and authoritative bodies varies based on their different rational value orientations.

The value proposition of scientific rationality is closely related to the foundations of institutional trust. It inherently recognizes the authority of expertise and public institutions and trusts them for evidence-based risk management of GM food.^52,74^ As a result, individuals who adhere to scientific rationality are more likely to exhibit higher levels of trust in existing GM technology governance systems, such as government-led risk regulation.^54,75^ In contrast, social rationality is critical of institutional trust. It questions the concentration of power and monopoly of knowledge in technological decision-making, and advocates for the democratization of decision-making and the incorporation of different values.^17,61^ As a result, individuals guided by social rationality typically exhibit lower levels of trust in authorities that govern GM technology (e.g., government regulators) and shift their trust to unofficial actors that better represent social justice and public participation.^45,76^ Rooted in fundamental ethical principles, ecological rationality provides a more in-depth value review of the institutional frameworks that underpin technological progress. It argues that existing risk governance paradigms (e.g., the principle of “substantial equivalence”) prioritize economic interests in their hierarchy of values, while systematically ignoring the intrinsic value of the integrity of nature and life.^67,68^ As a result, individuals upholding ecological rationality tend to perceive current GM regulatory authorities as failing to fulfill their core environmental protection and ethical oversight responsibilities, which directly contributes to low levels of institutional trust.^77–79^

Based on the above theoretical reasoning, this study proposes the following hypotheses regarding the relationship between the rationality framework and institutional trust:

H8: Different types of rationality predict institutional trust.

H8.a: Scientific rationality positively predicts institutional trust.

H8.b: Social rationality negatively predicts institutional trust.

H8.c: Ecological rationality negatively predicts institutional trust.

## 3. Methods

### 3.1. Participants and Procedures

The data for this study came from a questionnaire survey conducted in mainland China. In order to effectively reflect the socio-economic and cultural heterogeneity of different regions in China, a geographically stratified sampling strategy was employed. Three regional central cities were selected as survey sites: Shanghai (representing the developed eastern coastal region), Wuhan (representing the central region), and Chengdu (representing the western region). These cities are located within the Yangtze River Delta, the Middle Yangtze River, and the Chengdu-Chongqing Economic Zones, respectively. Their gradient differences in economic development stages, urbanization levels, and public awareness of technology collectively constitute an analytical sample that reasonably captures the diversity of China’s urban population.

In each city, 400 paper-based questionnaires were distributed through random intercept interviews in different locations, including commercial districts, residential areas, and public leisure spaces. Prior to the survey, all participants were fully informed of the study’s purpose and confirmed their participation based on informed consent and voluntary principles. In particular, before distributing the questionnaires, we conducted one-on-one explanations about genetically modified technology and related matters with each respondent, so that each respondent could understand that the genetically modified technology involved in this survey is a technique that uses biotechnological means to introduce specific exogenous genes into the genome of the target organism, enabling it to acquire new and stably inheritable traits. Genetically modified food refers to agricultural products and processed products made from crops, livestock, etc., that have acquired new traits such as resistance to pests and tolerance to herbicides through the above-mentioned gene modification technology, and are then available for direct human consumption or processing and consumption as raw materials. We solemnly promised the anonymity and confidentiality of the data and informed participants that they could withdraw from the survey at any time. A total of 1200 questionnaires were distributed. After rigorous data cleaning, we excluded invalid responses due to incomplete answers, systematic response patterns (e.g., straight-line checking), or obvious logical inconsistencies. This yielded 1020 valid questionnaires, representing an effective response rate of 85%. Table 1 presents the demographic characteristics of the final sample. As shown, the sample exhibits a wide range of differences in key demographic variables, including gender, age, educational level, and monthly personal income, capturing a broad range of demographic characteristics within China’s urban population.

**Table 1.** Demographic characteristics of respondents.

Variables	Category	Frequency	Percentage(%)
Gender	Male	502	49.22
	Female	518	50.78
Age	19 or below	96	9.41
	20–30	208	20.39
	31–40	278	27.25
	41–50	242	23.73
	51–60	107	10.49
	61 or above	89	8.73
Education	High school or below	369	36.18
	College or University	538	52.74
	Master or above	113	11.08
Income	5000 yuan or below	252	24.71
	5001–10000 yuan	467	45.78
	10001–20000 yuan	195	19.12
	20001 yuan or above	106	10.39

### 3.2. Measures

This study utilizes quantitative research methodology. A questionnaire was rigorously designed based on the theoretical framework shown in Figure 1. The first section collected demographic information through multiple-choice questions, including gender, age, education level, and income. All other core constructs were measured using a 7-point Likert scale (1 = “strongly disagree” to 7 = “strongly agree”). Public attitudes were measured using three question items adapted from Bredahl,^80^ with wording fine-tuned to the current research context. Perceived benefits and perceived risks were measured using four question items each, adapted from Siegrist^81^ conceptual framework and rephrased to fit the specific domain of GM food. We aimed to combine social interests with individual interests, and social risks with individual risks. For instance, we not only pay attention to the personal benefits of genetically modified foods, such as improving food taste and increasing income, but also reflect social benefits like reducing food shortages and soil pollution in the items. Institutional trust was measured using four question items developed by the authors based on the theoretical discussion of Pang,^82^ which conceptualizes trust in technical regulatory agencies in the Chinese context. Scientific rationality and social rationality do not have readily available measurement scales in the literature, but Isaac’s study^11^ outlines the characteristics of these two rationalities in the GM debate, on which we developed four items for each construct. Ecological rationality was measured using a four-item scale developed by the authors based on Todd et al.‘s conceptual framework,^83^ which emphasizes ethical and environmental concerns in technology assessment. Before the start of the large-scale survey, all questions were pretested with a small sample (*N* = 50) to ensure clarity, relevance, and comprehensibility. The results of a follow-up confirmatory factor analysis (CFA) supported the factorial validity and reliability of all scales. See Table 2 for details of the specific questions.

**Table 2.** Constructs and items included in the questionnaire.

Constructs	Items	Labels
Public attitudes	GM food is extremely bad- extremely good.	PA_1_
	GM food is extremely foolish - extremely wise.	PA_2_
	I am strongly against - strongly for GM food.	PA_3_
Perceived benefits	GM food will increase the nutrition of products, and improve the taste of food.	PB_1_
	GM food can reduce costs and increase farmers’ income.	PB_2_
	GM food can be helpful in addressing food shortages.	PB_3_
	GM food can help reduce the use of pesticides and fertilizers, thereby reducing pollution to water and soil.	PB_4_
Perceived risks	GM food is harmful to health, and products may be toxic and carcinogenic.	PR_1_
	GM food leads to the control of the seed market by other countries, thus threatening national security.	PR_2_
	GM food is harmful to biodiversity.	PR_3_
	GM food threatens the natural order.	PR_4_
Scientific rationality	The risk of GM food is objective, calculable and controllable.	SCR_1_
	The benefits of products, markets, and wealth are the primary focus of GM food applications.	SCR_2_
	The decision-making associated with GM food depends on expert systems.	SCR_3_
	The risk governance related to GM food should be based on scientific evidence as the fundamental principle.	SCR_4_
Social rationality	The risk of GM food is uncalculated, unattributable and socially constructive.	SOR_1_
	Uncertain consequences are the primary characteristic of GM food applications.	SOR_2_
	The decision-making associated with GM food depends on both expert and civic participation.	SOR_3_
	The risk governance related to GM food should be based on the precautionary principle.	SOR_4_
Ecological rationality	The risk of GM food is an issue of ecological ethics.	ECR_1_
	Inter-generational environmental consequences are the primary concern in GM food applications.	ECR_2_
	The decision-making associated with GM food depends on civic participation.	ECR_3_
	The risk governance related to GM food should be based on the principle of ethical acceptability.	ECR_4_
Institutional trust	I believe regulatory authorities have the capability to govern risks associated with GM food.	IT_1_
	I believe regulatory authorities act with integrity in matters related to GM food.	IT_2_
	I believe regulatory authorities act in the public’s best interest regarding GM food.	IT_3_
	I believe the regulatory system for GM food is effective.	IT_4_

### 3.3. Data Analysis Procedure

Structural Equation Modeling (SEM) was used as the primary analytical method in this study, and a standard three-step procedure was followed. First, descriptive statistical analysis was conducted on all core variables to characterize their basic distributions. Second, Confirmatory Factor Analysis (CFA) was performed to examine the reliability and validity of the measurement model, ensuring the psychometric soundness of the instrument. Finally, the model was estimated using AMOS software to assess the overall model goodness-of-fit and the significance of each hypothesized path.

Structural Equation Modeling is a multivariate statistical technique that integrates factor analysis and path analysis and is able to simultaneously address complex relationships between observed and latent variables. This approach is particularly suitable for testing the theoretical framework proposed in this study, as it is capable of simultaneously estimating direct and indirect effects (including mediation) between multiple independent and dependent variables. In addition, given that the formation of public attitudes toward GM food involves a complex interplay between multiple rationalities, institutional trust, and risk-benefit perceptions, SEM provides a rigorous empirical framework for analyzing this complex mechanism.

## 4. Results

### 4.1. Descriptive Analysis

Before testing the measurement and structural models, this study first conducted descriptive statistical analyses of all core variables, and the results are presented in Table 3. The data indicate that the overall attitude score of the Chinese public toward GM food is relatively low (*M* = 3.30), which is lower than the scale midpoint of 4. This indicates that respondents’ attitudes toward GM food were generally negative. Further analysis showed that although the public’s perception of the benefits of GM food was at a moderate level (*M* = 4.30), the risk perception was considerably higher (*M* = 4.73). This cognitive pattern of risk perception exceeding benefit perception may be a key antecedent associated with their negative attitudes.

**Table 3.** Descriptive statistical results.

Variables	Means	Standard Deviation
Public Attitudes	3.30	1.17
Perceived Benefits	4.30	1.25
Perceived Risks	4.73	1.46
Scientific Rationality	3.48	0.94
Social Rationality	4.11	1.26
Ecological Rationality	4.42	1.17
Institutional Trust	4.09	1.02

In terms of the rationality framework, the data analysis revealed a clear trend: the public scored relatively high on ecological rationality (*M* = 4.42) and social rationality (*M* = 4.11) compared to scientific rationality (*M* = 3.48) when evaluating GM food. This finding provides initial empirical support for the basic premise of this study that values and ethical concerns play a key role in public risk decision-making. In addition, institutional trust is close to the midpoint of the scale (*M* = 4.09), reflecting a generally neutral attitude toward the regulatory agencies. This provides a valuable analytical foundation for subsequent research on the potential role of institutional trust in theoretical modeling.

Table 4 presents the group variance analysis of public attitudes. The results show that there are no significant differences in the public’s attitudes toward genetically modified foods in terms of gender, age, and income. However, there is a significant difference in the education level dimension. The more educated the public is, the more positive their attitude toward genetically modified foods will be.

**Table 4.** Results of group difference analysis across demographic characteristics.

	Variables	Category	Means	Standard Deviation	T/F
Public Attitudes	Gender	Male	3.32	1.15	−0.56
		Female	3.28	1.12	
	Age	19 or below	3.25	1.13	0.25
		20–30	3.26	1.19	
		31–40	3.32	1.18	
		41–50	3.28	0.96	
		51–60	3.35	1.12	
		61 or above	3.37	0.99	
	Education	High school or below	3.08	0.89	13.26***
		College or University	3.39	1.21	
		Master or above	3.58	1.01	
	Income	5000 yuan or below	3.27	0.97	0.23
		5001–10000 yuan	3.29	1.15	
		10001–20000 yuan	3.32	1.19	
		20001 yuan or above	3.37	1.13	

Note. For two-group comparisons (gender), *t*-values are reported. For comparisons involving three or more groups (age, education, income), F-values are reported. Unmarked F-values are not statistically significant (*p* > .05). *p < .05. **p < .01. ***p < .001.

### 4.2. Measurement Model Assessment

To ensure the validity of the structural equation modeling (SEM) analysis, this study first conducted reliability and validity tests on the measurement model, with results summarized in Table 5. The reliability analysis indicated that the composite reliability (CR) for all constructs exceeded 0.80, Cronbach’s α coefficients were all greater than 0.70, and the corrected item-total correlation (CITC) exceeded the recommended threshold of 0.70. These metrics consistently indicate that the measurement model possesses satisfactory internal consistency. Regarding validity, all standardized factor loadings of the measurement items surpassed 0.70, and the average variance extracted (AVE) for each construct surpassed the discriminant criterion of 0.50, providing strong evidence for convergent validity.

**Table 5.** Reliability and validity test results.

Construct	Items	Loadings	CITC	CR	Cronbach’s *α*	AVE
SCR	SCR_1_	0.85***	0.72	0.95	0.83	0.95
	SCR_2_	0.90***	0.74			
	SCR_3_	0.75***	0.71			
	SCR_4_	0.83***	0.72			
SOR	SOR_1_	0.72***	0.70	0.89	0.84	0.90
	SOR_2_	0.84***	0.75			
	SOR_3_	0.75***	0.72			
	SOR_4_	0.80***	0.73			
ECR	ECR_1_	0.87***	0.75	0.92	0.87	0.85
	ECR_2_	0.91***	0.81			
	ECR_3_	0.91***	0.81			
	ECR_4_	0.89***	0.75			
PB	PB_1_	0.81***	0.75	0.94	0.88	0.80
	PB_2_	0.80***	0.72			
	PB_3_	0.82***	0.75			
	PB_4_	0.79***	0.76			
PR	PR_1_	0.82***	0.83	0.97	0.92	0.85
	PR_2_	0.86***	0.77			
	PR_3_	0.81***	0.77			
	PR_4_	0.89***	0.79			
PA	PA_1_	0.81***	0.75	0.92	0.85	0.92
	PA_2_	0.88***	0.76			
	PA_3_	0.81***	0.72			
IT	IT_1_	0.81***	0.83	0.90	0.84	0.94
	IT_2_	0.77***	0.77			
	IT_3_	0.77***	0.77			
	IT_4_	0.78***	0.79			

Note. *p < .05. **p < .01. ***p < .001.

Furthermore, as shown in the diagonal elements in Table 6, the square roots of the AVE for each construct were greater than their absolute correlations with any other construct, satisfying the Fornell-Larcker criterion and indicating good discriminant validity of the measurement model. In summary, the measurement model of this study meets the required standards for both reliability and validity, thereby laying a solid foundation for the subsequent analysis of the structural model.

**Table 6.** Correlation matrix and discriminant validity assessment.

Construct	SCR	SOR	ECR	PB	PR	PA	IT
SCR	**0.97**						
SOR	−0.47***	**0.95**					
ECR	−0.36**	0.60**	**0.92**				
PB	0.13**	−0.22**	−0.23**	**0.92**			
PR	−0.12**	0.22***	0.23***	−0.22**	**0.90**		
PA	0.10**	−0.24***	−0.23***	0.36***	−0.40***	**0.96**	
IT	0.28**	−0.49**	−0.57**	0.30**	−0.23**	0.44**	**0.97**

Note: *p < .05. **p < .01. ***p < .001; bold diagonal elements are square roots of the average variance extracted (√AVE).

### 4.3. Structural Model and Hypothesis Testing

The overall goodness-of-fit results for the Structural Equation Model (SEM) are summarized in Table 7. The analysis indicates that the model fits the data well, with all key metrics meeting or exceeding common thresholds: the normed chi-square (*x*^*2*^ */df*) is 3.43, below the liberal benchmark of 5.0; the Root Mean Square Error of Approximation (RMSEA) was 0.05, meeting the good fit threshold below 0.08; the Comparative Fit Index (CFI), Tucker-Lewis Index (TLI), and Normed Fit Index (NFI) were 0.92, 0.92, and 0.90, respectively, all exceeding the recommended standard of 0.90; The Goodness-of-Fit Index (GFI) and its adjusted version (AGFI) were 0.90 and 0.91, also meeting model fit requirements. Furthermore, the parsimonious fit indices, including PGFI, PNFI, and PCFI, all exceeded the acceptable threshold of 0.50. A comprehensive analysis indicates that although the *x*^*2*^ */df* slightly exceeded the stricter 3.0 criterion, considering this metric’s sensitivity to large sample sizes and the robust performance of all other fit indices, the model demonstrates good fit with the observed data. This validated model was therefore deemed suitable for subsequent testing of path coefficients and hypothesis analysis.

**Table 7.** Goodness-of-fit statistics for structural model.

Index		Threshold	Acceptance
*x*^*2*^ */df*	3.43	<5.0	Passed
RMSEA	0.05	<0.08	Passed
CFI	0.92	>0.9	Passed
TLI	0.92	>0.9	Passed
NFI	0.90	>0.9	Passed
GFI	0.90	>0.9	Passed
AGFI	0.91	>0.9	Passed
PGFI	0.51	>0.5	Passed
PNFI	0.51	>0.5	Passed
PCFI	0.56	>0.5	Passed

In terms of explanatory power, the structural model explained a significant proportion of the variance in the key endogenous variables. Specifically, the model explained 53.6% of the variance in public attitudes (R^2^ = 0.536), 40.1% of the variance in perceived risks (R^2^ = 0.401), 39.7% of the variance in perceived benefits (R^2^ = 0.397), and 38.6% of the variance in institutional trust (R^2^ = 0.386). These values indicate that the proposed theoretical framework has satisfactory explanatory power for understanding the formation of public attitudes toward GM food.

**Figure 2.**
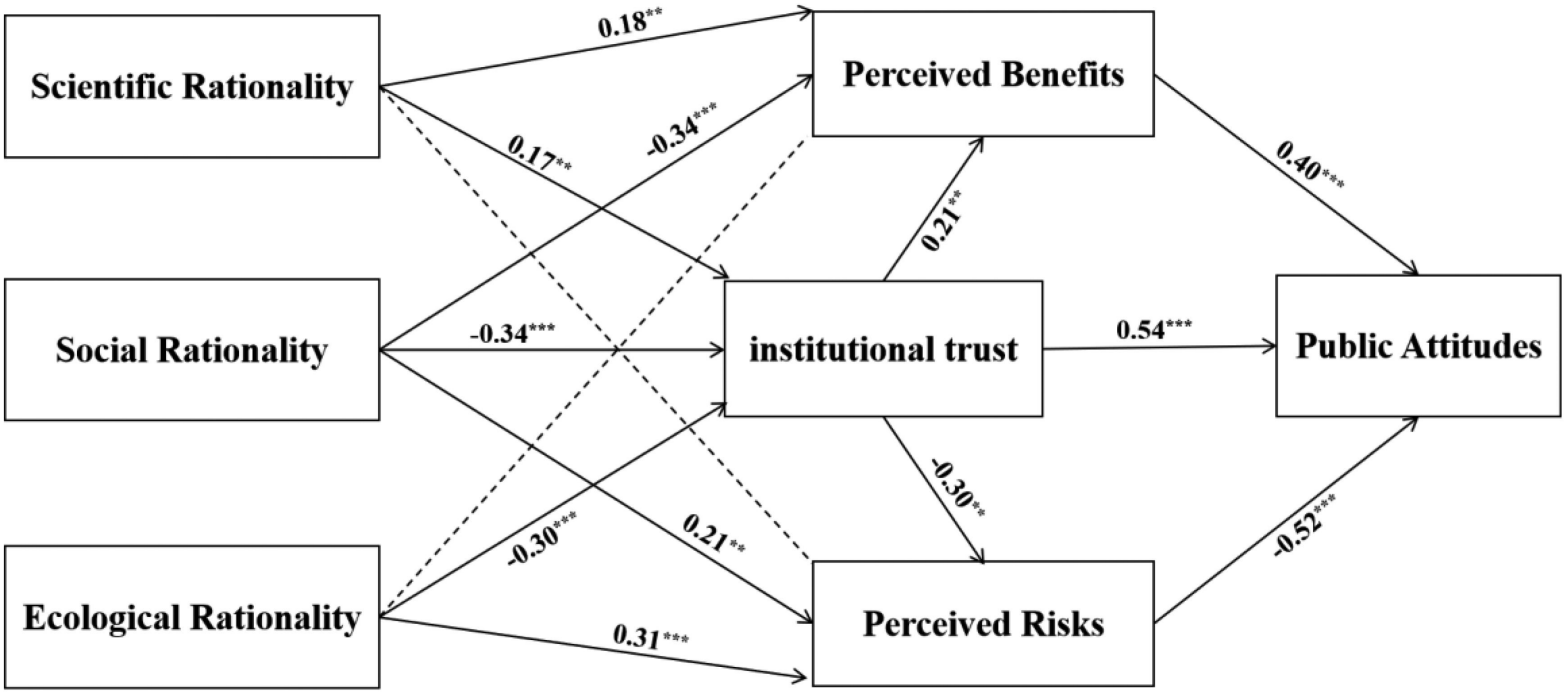
Results of structural model.

Based on the results of testing the theoretical model using AMOS software, most of the hypotheses proposed in this study received empirical support. As shown in Table 8 and Figure 2, among the 14 hypothesized paths in the model, 12 path coefficients were statistically significant and their directions of associations aligned with theoretical expectations. This outcome not only confirms the robustness of the core variable relationships in the conceptual model but also verifies the model’s overall explanatory power.

**Table 8.** Results of structural Equation modeling.

Hypothesis	Hypothesized Relationships			Estimate	SE	P	Results
H1	PA	<—	PB	0.40	0.09	<0.001	Supported
H2	PA	<—	PR	−0.52	0.08	<0.001	Supported
H3	PB	<—	IT	0.21	0.09	<0.01	Supported
H4	PR	<—	IT	−0.29	0.09	<0.01	Supported
H5	PA	<—	IT	0.54	0.06	<0.001	Supported
H6.a	PB	<—	SCR	0.18	0.10	<0.01	Supported
H6.b	PB	<—	SOR	−0.34	0.10	<0.001	Supported
H6.c	PB	<—	ECR	−0.06	0.07	0.107	No
H7.a	PR	<—	SCR	−0.08	0.11	0.639	No
H7.b	PR	<—	SOR	0.21	0.11	<0.01	Supported
H7.c	PR	<—	ECR	0.31	0.09	<0.001	Supported
H8.a	IT	<—	SCR	0.17	0.04	<0.01	Supported
H8.b	IT	<—	SOR	−0.34	0.04	<0.001	Supported
H8.c	IT	<—	ECR	−0.30	0.09	<0.001	Supported

The results of the path analysis showed that perceived benefits positively predicted public attitudes toward GM food (β = 0.40, *p* < .001), supporting H1. However, perceived risks had a significant negative effect on attitudes (β = −0.52, *p* < .001), confirming H2. This suggests that the formation of public attitudes toward GM food follows the Risk-benefit Analysis Framework. Regarding the specific pathway of institutional trust, it not only positively predicted perceived benefits (β = 0.21, *p* < .01, supporting H3) but also negatively affected perceived risks (β = −0.29, *p* < .01, supporting H4). In addition, institutional trust also had a significant direct positive effect on public attitudes (β = 0.54, *p* < .001, supporting H5). Overall, these findings emphasize the central role of institutional trust in influencing public attitudes.

Further analysis showed that there were different influence paths for the three types of rationality. Scientific rationality significantly positively predicted perceived benefits (β = 0.18, *p* < .01, confirmed by H6.a), while the effect on perceived risk was not significant (not confirmed by H7.a). Social rationality significantly reduced perceived benefits (β = −0.34, *p* < .001, supported by H6.b) while increasing perceived risk (β = 0.21, *p* < .01, supported by H7.b). Ecological rationality primarily had a significant positive effect on perceived risk (β = 0.31, *p* < .001, supporting H7.c), while the effect on perceived benefits was not statistically significant (not supporting H6.c). This pattern reflects how different types of rationality influence public perceptions through different pathways: scientific rationality is linked to positive attitudes by increasing perceptions of benefits; social rationality is linked to negative attitudes by suppressing perceptions of benefits and increasing perceptions of risk; and ecological rationality is linked to negative attitudes primarily by reinforcing perceptions of risk.

In addition, the rationality framework had significant but different effects on institutional trust. Scientific rationality positively predicted institutional trust (β = 0.17, *p* < .01), supporting H8.a. In contrast, social rationality (β = −0.34, *p* < .001) and ecological rationality (β = −0.30, *p* < .001) both exhibited significant negative effects, thus validating H8.b and H8.c. These findings suggest that individuals’ rational orientation is largely associated with the basis of their trust in institutions: those who recognize scientific authority are more inclined to trust public institutions, whereas those motivated by considerations of social justice or natural ethics tend to express a stronger distrust of technological governance systems.

To further elucidate the mechanisms by which pluralistic rationality is associated with public attitudes, we conducted 5,000 resampled Bootstrapping analyses to test for indirect effects through institutional trust and perceived risks/benefits. Scientific rationality had a significant positive indirect effect on attitudes through the sequential paths of institutional trust and perceived benefits (β = 0.02, 95% CI [0.00, 0.03]), but its indirect effect through institutional trust and perceived risk was not significant (β = 0.01, 95% CI [−0.00, 0.05]), suggesting that the influence of scientific rationality was realized mainly through the benefits path. Social rationality had a significant negative indirect effect on attitudes through both the institutional trust-perceived benefit path (β = −0.03, [−0.04, −0.02]) and the institutional trust-perceived risk path (β = −0.05, [−0.08, −0.02]). Ecological rationality had a significant negative indirect effect through the institutional trust-perceived risk path (β = −0.04, [−0.07, −0.02]). The three types of rationality also had significant indirect effects on attitudes through institutional trust alone: scientific rationality positive (β = 0.10, [0.04, 0.15]), social rationality negative (β = −0.19, [−0.25, −0.13]), and ecological rationality negative (β = −0.17, [−0.23, −0.11]). In the path without institutional trust, scientific rationality had a significant positive indirect effect on attitudes through perceived benefits (β = 0.08, [0.04, 0.12]). Social rationality had a significant negative indirect effect on attitudes through both perceived benefits (β = −0.14, [−0.19, −0.08]) and perceived risks (β = −0.12, [−0.17, −0.06]). Ecological rationality had a significant negative indirect effect through perceived risk (β = −0.17, [−0.23, −0.11]). Taken together, the mediation effect results suggest that institutional trust and risk/benefit perception are key mechanisms linking pluralistic rationality to public attitudes. The pattern of indirect effects is highly consistent with the direct path coefficients, validating the robustness of the model.

## 5. Discussion

### 5.1. Theoretical Implications

The Theory of Planned Behavior (TPB) offers a general framework for understanding attitudes and behaviors, but it assumes behavioral beliefs as given and does not explain their origins. To address this gap, this study introduces the rationality framework into TPB, positioning scientific, social, and ecological rationality as the deep value orientations that shape these beliefs. By specifying where belief differences come from, this study extends the TPB in two ways: first, by explaining the origins of the beliefs that TPB takes as exogenous; second, by incorporating institutional trust as a mediator between value orientations and risk/benefit perceptions, thus refining the TPB’s account of how external factors are processed through the subjective value system. This study focuses on examining value-driven pathways within the TPB, thereby providing a complementary perspective to existing research on heuristic and affective influences.^27,84^

The results of this study are consistent with the Risk-benefit Analysis Framework^28,81^: perceived benefits have a significant positive effect on public attitudes, while perceived risks have a significant negative effect. However, this difference in coefficients needs to be interpreted with caution. Bearth and Siegrist’s meta-analysis found that the predictive weights of risk and benefit perceptions were roughly equal in several studies on innovative food technologies,^85^ and there was no consistent evidence of a “negative bias^86,87^.” The slightly higher risk pathway coefficients observed in this study may be related to the China-specific context, where GM food has long been under public controversy and media spotlight.^4,88^ This environment may have reinforced the public’s risk sensitivity,^89^ giving greater weight to risk perceptions in decision-making trade-offs. The explanatory power of the model for public attitudes showed that after integrating the rationality framework and institutional trust into the TPB and the Risk-benefit Analysis Framework, the model was able to provide a reliable empirical basis for revealing the pathways of attitudes toward GM food.

As the deep value structures that shape belief systems, the rationality framework influences public attitudes through differentiated pathways. Scientific rationality, characterized by an emphasis on verifiable evidence, expert authority, and technological efficacy,^53^ positively predicted perceived benefits but was not significantly associated with perceived risks. This suggests that individuals with this value orientation tend to focus on the instrumental aspects of GM food and believe that risks can be managed through expert-led regulation.^16,90^ Social rationality focuses on power distribution, procedural justice, and public participation,^61^ negatively predicting perceived benefits and positively predicting perceived risks. This dual pattern corroborates previous research findings that individuals who question the fairness of technological governance are more likely to scrutinize the distribution of benefits and potential harms.^58,91^ Ecological rationality, which is rooted in ethical concerns about naturalness and the intrinsic value of life,^67^ positively predicted perceived risks but was not significantly associated with perceived benefits. This finding supports the view that, for some individuals, opposition to GM food is not based on utilitarian calculations but rather stems from an ethical stance that views genetic modification as contrary to the natural order.^92,93^ Together, these differentiated pathways reveal that public attitudes are not only the result of knowledge deficits or rational calculations, but also reflect deep value commitments that direct attention to different dimensions of the technology.

The findings further highlight the mediating role of institutional trust in this value-driven cognitive process. Trust positively predicts perceived benefits and public attitudes and negatively predicts perceived risks.^94^ Analysis of mediating effects revealed that trust was the key mechanism linking the rationality framework to final attitudes. The indirect path from scientific rationality to attitudes via institutional trust and perceived risk was not significant, while the path via perceived benefits was significant. This pattern reveals that individuals upholding scientific rationality develop positive attitudes primarily through enhanced perceived benefits, in which trust plays a facilitating role, as opposed to through reduced risk perceptions. In other words, for scientifically rational individuals, beliefs about the risk controllability may make the correlation between risk paths and attitude formation weaker. Social rationality and ecological rationality provide further evidence that these value orientations are influenced through different psychological mechanisms through significant indirect effects of trust and risk/benefit perceptions.^31,48^ However, there is an ongoing controversy in the literature regarding the causal direction of trust and risk/benefit perceptions: some studies view trust as an antecedent of risk and benefit perceptions,^95–98^ while others propose that trust may be a consequence of perceived value congruence or a consequence of prior risk assessment.^10,99^ Limited by the cross-sectional design, the present study was unable to make judgments between these competing causal explanations, but the data reveal a systematic and intrinsic association between trust, risk/benefit perceptions, and value orientation.

### 5.2. Practical Implications

Based on these findings, risk communication needs to be differentiated for different rationality groups. For the scientific rationality group, this study reveals that their attitudes are mainly formed through benefit perceptions. Their reliance on institutional trust is reflected in the indirect path, indicating that this group exhibits higher levels of institutional trust. Therefore, communication to this group should focus on highlighting scientific evidence and technological benefits while conveying the effectiveness of the regulatory system. The socially rational group exhibits a dual cognitive pattern: they have a lower perception of benefits, a higher perception of risk, and a lower level of institutional trust. To address their core demands for power distribution and procedural justice, the focus of communication should go beyond the mere transmission of information to the transparency of the decision-making process and the establishment of public participation mechanisms. The ecological rationality group forms its attitudes mainly through risk perceptions and also has a low level of trust in institutions. For this group, communication must extend beyond utilitarian calculations to a genuine concern for their ethical and ecological considerations, emphasizing rigorous ethical review and robust ecological protection mechanisms.

The risk communication of genetic modification technology should go beyond the limitations of a single rational dimension, integrating the credibility of scientific rationality, the fairness of social rational participation, and the long-term limits of ecological rationality into an organic whole. Communication should abandon one-way authoritative declarations and instead openly disclose the methodology of risk assessment, the sources of data, and the boundaries of uncertainty, enabling the public to establish their own judgments during the process of understanding the formation of scientific consensus; at the same time, a two-way dialogue mechanism should be constructed to absorb the voices of farmers, consumers, environmental organizations, etc. at the decision-making front, responding to the doubts about the fairness of the distribution of technological benefits and risks; even more importantly, it is necessary to openly face the ecological uncertainties such as gene flow and biodiversity, incorporate the principle of prevention into the public discussion framework, and allow short-term gains and long-term costs to be weighed against each other. Only when the three kinds of rationality are mutually checked and supplemented in the negotiation and dialogue, can risk communication break free from the predicament of each speaking their own language, and sincerely respond to the deep concerns of the public regarding the safety of the technology, social justice, and ecological sustainability, thereby facilitating a dynamic and responsible collective judgment in the context of diverse value tensions.

Institutional trust should be a systemic goal of technology governance. This study reveals that institutional trust is not only a direct predictor of attitudes but also a key hub connecting value orientations and risk/benefit perceptions. This implies that even if communication strategies are well-designed, without a solid foundation of trust, it may be difficult to achieve the desired results. The mandatory labeling policy plays a crucial role in strengthening the institutional trust of the public in genetically modified technology. The government must grant the public the right to know and the right to choose by mandatorily disclosing information, thereby breaking the chain of suspicion known as “regulation and industry collusion” and preventing trust from being continuously eroded. At the same time, the labeling system itself, as a formal signal of government active supervision, indicates to the public that genetically modified technology has been incorporated into an open and transparent public governance framework. This “being seen and being managed” institutionalized state can alleviate the unease associated with the technology.

Therefore, the public institutions need to establish long-term, transparent, and accountable decision-making mechanisms that substantively incorporate the value concerns of different rationality groups into the institutional framework. For example, the introduction of ethical review committees in the risk assessment process, the establishment of a risk communication platform with public participation, and the regular release of transparent regulatory reports. Only when trust becomes a systemic goal of technology governance, rather than a remedial measure after a crisis occurs, can the above-mentioned differentiated communication and value dialogue be truly effective.

### 5.3. Limitations and Future Research

This study has several limitations, which provide directions for future research improvement. At the methodological level, this study used cross-sectional data, which revealed variable associations but did not establish causality. Future research may manipulate the level of trust through experimental design or reveal the temporal order of the variables through longitudinal tracing to clarify these relationships more clearly.

Firstly, this study has certain limitations in sample selection. The survey only covered three cities: Shanghai, Wuhan, and Chengdu. Although the research design attempted to treat these three cities as typical representatives of the eastern, central, and western regions respectively, the sample was completely limited to urban residents and did not include rural populations, resulting in a significant coverage bias. Rural areas have systematic differences in terms of information accessibility, educational level, technological contact experience, and the daily reliance on government regulatory systems compared to urban residents. Their perception of the risks and benefits of emerging technologies may follow different generation logic. In particular, whether institutional trust still plays the same “simplification mechanism” in informationally closed or knowledge-dominated rural fields, and whether the blunting state of knowledge cognition pathways is more significant or shows an opposite trend, all require empirical verification. Therefore, future research needs to include multi-level and multi-regional mixed samples, especially focusing on the comparative design of rural and urban samples, in order to enhance the breadth and effectiveness of theoretical explanations.

Secondly, this study shows a lack of attention to the dimension of stakeholders. The survey subjects were strictly limited to the general public, and the government regulatory authorities, technology enterprises, social organization practitioners, and farmers, who have clear functional positioning and interest demands, were not included in the analytical framework. The differentiation of stakeholder roles may have determined the foundation for their construction of institutional trust and the logic of assigning weights to risks and benefits. This is bound to have essential differences from ordinary urban residents who lack institutional internal experience and professional technical background. Future research is necessary to adopt a multi-agent comparative design to systematically examine the heterogeneity of the model’s effects among different stakeholders, thereby providing a more inclusive correction and development space for the risk perception theory.

Furthermore, at the level of theoretical depth, this study focuses on the subjective value (the rationality framework), recognizing emotion and heuristic process as contextual factors but not directly measuring them. These factors can be integrated to examine their interaction with the rationality framework in the future. At the level of rationality formation mechanisms, this study used the rationality framework as antecedent variables, but did not delve into the formation of these value orientations themselves. Factors such as media exposure, cultural values, and educational experiences may shape individual rationality tendencies, and future research could trace the socialization and psychological origins of these rationalities to provide a more fundamental theoretical basis for early public participation and value-sensitive technology design.

## 6. Conclusion

This study constructed and tested a comprehensive model integrating the rationality framework and institutional trust, aiming to reveal the formation process of the Chinese public attitudes toward GM food. The results indicate that attitude formation follows a structured path from deep value orientation to trust judgment, to risk/benefit perception, and finally to attitudes. Specifically, three types of rationality influence attitudes in a differentiated way: scientific rationality is associated with positive attitudes mainly through enhanced perceived benefits; social rationality is associated with negative attitudes through the dual paths of suppressed perceived benefits and enhanced perceived risks; and ecological rationality is associated with negative attitudes mainly through amplified perceived risks. Institutional trust plays a key mediating role, not only directly predicting public attitudes, but also connecting subjective value orientations to risk/benefit perceptions. Notably, the indirect path from scientific rationality via trust and perceived risk is not significant, suggesting that this group’s positive attitudes stem primarily from a focus on benefits rather than a weakening of risk. The model explains 53.6% of the variance in public attitudes, confirming the utility of extending the TPB framework by specifying the origins of belief differences, namely the three types of rationality as deep value structures, and by incorporating institutional trust as a key mediating mechanism. These findings provide clear practical implications for risk management: communication strategies need to be differentiated by matching the value logics of different rationality groups; the scientific community should fully participate in the value dialogue; and institutional trust should be established as a systemic goal of technology governance. Future research could use longitudinal designs to examine causal direction, integrate affective measures to explore interaction with rationality pathways, and trace the role of media, culture, and education in shaping the rationality framework itself.

## Data Availability

The datasets generated and/or analyzed during the current study are not publicly available due to ongoing research utilizing the data, as well as privacy and confidentiality commitments made to participants. Access to the data may be granted by the corresponding author upon reasonable request and subject to a data-sharing agreement.

## References

[1] Ewa W-G, Agata T, Milica P, Anna B, Dennis E, Nick V, Godelieve G, Selim C, Naghmeh A, Tomasz T. Public perception of plant gene technologies worldwide in the light of food security. GM Crops Food. 2022;13(1):218–768. doi: 10.1080/21645698.2022.2111946. 35996854 PMC9415543

[2] Boccia F, Punzo G. A choice experiment on Consumer perceptions of three generations of genetically modified foods. Appetite. 2021;161:105158. doi: 10.1016/j.appet.2021.105158. 33561496

[3] Del-Aguila-Arcentales S, Alvarez-Risco A, Rojas-Osorio M, Meza-Perez H, Simbaqueba-Uribe J, Talavera-Aguirre R, Mayo-Alvarez L, Espinoza-Ipanaque P, Davies NM, Yáñez JA. Analysis of genetically modified foods and consumer: 25 years of research indexed in scopus. J Agriculture Food Res. 2024;19:101594. doi: 10.1016/j.jafr.2024.101594.

[4] Jin Y, Schaub S, Tosun J, Wesseler J. Does China have a public debate on genetically modified organisms? a discourse network analysis of public debate on Weibo. Public Underst Sci. 2022;31(6):732–50. doi: 10.1177/09636625211070150. 35086388 PMC9344491

[5] Thompson PB. Food and agricultural biotechnology in ethical perspective. Cham: Springer; 2020.

[6] Fiske ST, Taylor SE. Social cognition: from brains to culture. Los Angeles (CA): Sage; 2017.

[7] Renn O. Risk governance : coping with uncertainty in a complex world. London: Earthscan; 2010.

[8] Slovic P. Trust, emotion, sex, politics, and science: surveying the risk-assessment battlefield. Risk Anal. 1999;19(4):689–701. doi: 10.1111/j.1539-6924.1999.tb00439.x. 10765431

[9] Sleboda P, Lagerkvist C-J. Tailored communication changes consumers’ attitudes and product preferences for genetically modified food. Food Qual Preference. 2022;96:104419. doi: 10.1016/j.foodqual.2021.104419.

[10] Poortinga W, Pidgeon NF. Trust in risk regulation: cause or consequence of the acceptability of GM food? Risk Anal: Off Publ Soc Risk Anal. 2005;25(1):199–209. doi: 10.1111/j.0272-4332.2005.00579.x. 15787769

[11] Isaac GE. Gmos at the WTO—A harvest of trouble. J World Trade. 2003;37(Issue 6):1083–95. doi: 10.54648/trad2003056.

[12] Mallinson L, Russell J, Cameron DD, Ton J, Horton P, Barker ME. Why rational argument fails the genetic modification (GM) debate. Food Sec. 2018;10(5):1145–61. doi: 10.1007/s12571-018-0832-1.

[13] Li C, Li Y. Factors influencing public risk perception of emerging technologies: a meta-analysis. Sustainability. 2023;15(5):3939. doi: 10.3390/su15053939.

[14] Rihn A, Khachatryan H, Wei X. Perceived subjective versus objective knowledge: consumer valuation of genetically modified certification on food producing plants. PLOS ONE. 2021;16(8):e0255406. doi: 10.1371/journal.pone.0255406. 34411110 PMC8376035

[15] Zhan J, Ma Y, Deng P, Li Y, Xu M, Xiong H. Designing enhanced labeling information to increase Consumer willingness to pay for genetically modified foods. Br Food J. 2020;123(1):405–18. doi: 10.1108/bfj-08-2019-0637.

[16] Thuy T, Yen T, Ngoc T. Impacts of knowledge and trust on Consumer perceptions and purchase intentions towards genetically modified foods. PLOS ONE. 2024;19(10):e0311257–0311257. doi: 10.1371/journal.pone.0311257. 39356695 PMC11446447

[17] Hu L, Liu R, Zhang W, Zhang T. The effects of epistemic trust and social trust on public acceptance of genetically modified food: an empirical study from China. Int J Environ Res Public Health. 2020;17(20):7700. doi: 10.3390/ijerph17207700. 33096931 PMC7593935

[18] Fishbein M. An Investigation of the relationships between beliefs about an object and the attitude toward that object. Hum Relat. 1963;16(3):233–39. doi: 10.1177/001872676301600302.

[19] Chaiken S. Heuristic versus systematic information processing and the use of source versus message cues in persuasion. J Pers Soc Psychol. 1980;39(5):752–66. doi: 10.1037/0022-3514.39.5.752.

[20] Chinn S, Hasell A. Uniquely disgusting? Physiological disgust and attitudes toward GM food and other food and Health technologies. J Sci Commun. 2021;20(7):A05. doi: 10.22323/2.20070205.

[21] Gaskell G, Allum N, Bauer M, Durant J, Allansdottir A, Bonfadelli H, Boy D, de Cheveigné S, Fjaestad B, Gutteling JM, et al. Biotechnology and the European public. Nat Biotechnol. 2000;18(9):935–38. doi: 10.1038/79403. 10973210

[22] Mielby H, Sandøe P, Lassen J. The role of scientific knowledge in shaping public attitudes to GM technologies. Public Underst Sci. 2012;22(2):155–68. doi: 10.1177/0963662511430577. 23833022

[23] Lupia A. Communicating science in politicized environments. Proc Natl Acad Sci USA. 2013;110(Supplement_3):14048–54. doi: 10.1073/pnas.1212726110. 23940336 PMC3752174

[24] Druckman JN, Bolsen T. Framing, motivated reasoning, and opinions about emergent technologies. J Commun. 2011;61(4):659–88. doi: 10.1111/j.1460-2466.2011.01562.x.

[25] Fishbein M, Ajzen I. Belief, attitude, intention, and behavior: an introduction to theory and research. Massachusetts: Addison-Wesley Pub. Co; 1975. https://people.umass.edu/aizen/f&a1975.html.

[26] Ajzen I. The theory of Planned Behavior. Organizational Behav Hum Decis Processes. 1991;50(2):179–211. doi: 10.1016/0749-5978(91)90020-T.

[27] Frewer LJ, van der Lans IA, Fischer ARH, Reinders MJ, Menozzi D, Zhang X, van den Berg I, Zimmermann KL. Public perceptions of agri-food applications of genetic modification – a systematic review and meta-analysis. Trends Food Sci Technol. 2013;30(2):142–52. doi: 10.1016/j.tifs.2013.01.003.

[28] Bredahl L, Grunert KG, Frewer LJ. Consumer attitudes and decision-making with regard to genetically engineered food products – a review of the literature and a presentation of models for future research. J Consum Policy (Dordr). 1998;21(3):251–77. doi: 10.1023/a:1006940724167.

[29] Vega Rodríguez A, Rodríguez-Oramas C, Sanjuán Velázquez E, Hardisson de la Torre A, Rubio Armendáriz C, Carrascosa Iruzubieta C. Myths and realities about genetically modified food: a risk-benefit analysis. Appl Sci. 2022;12(6):2861. doi: 10.3390/app12062861.

[30] Du Z, Xiao Y, Xu J. How does information exposure affect public attitudes toward GMO in China? the mediating and moderating roles of conspiracy belief and knowledge. Front Psychol. 2022;13:13. doi: 10.3389/fpsyg.2022.955541. PMC9530470 36204765

[31] Yang Z, Liao D, Jia H. Similarities and differences with the ‘general public’: Chinese civil servants’ attitude to genetically modified organisms and its influencing factors. GM Crops Food. 2023;14(1):1–13. doi: 10.1080/21645698.2023.2256929. PMC10503451 37707999

[32] Membré JM, Santillana Farakos S, Nauta M. Risk-benefit analysis in food safety and nutrition. Curr Opin Food Sci. 2021;39:76–82. doi: 10.1016/j.cofs.2020.12.009.

[33] Islam MS, Roy S, Alfee SL, Pal A. An empirical study of the risk-benefit perceptions between the nuclear and non-nuclear groups towards the nuclear power plant in Bangladesh. Nucl Eng Technol. 2023;55(12):4617–27. doi: 10.1016/j.net.2023.07.047.

[34] Visschers VHM, Feck V, Feck A. Knowledge, social influences, perceived risks and benefits, and cultural values explain the public’s decisions related to prudent antibiotic use. Risk Anal. 2021;42(7):1488–503. doi: 10.1111/risa.13851. 34784422 PMC9544676

[35] Ali M, Raza SA, Khamis B, Puah CH, Amin H. How perceived risk, benefit and trust determine user fintech adoption: a New dimension for Islamic finance. FS. 2021;23(4):403–20. doi: 10.1108/fs-09-2020-0095.

[36] Ocampo JC, Carter LM, Brown JL, Marquis H, Uribe CF, Zanzonico PB, Bolch WE, Kesner AL. The risk Index as a basis for risk/benefit analyses and protocol optimization in diagnostic nuclear imaging. Med Phys. 2023;50(12):7390–99. doi: 10.1002/mp.16696. 37656137 PMC12445442

[37] Prati G, Pietrantoni L, Zani B. The prediction of intention to consume genetically modified food: test of an integrated psychosocial model. Food Qual Preference. 2012;25(2):163–70. doi: 10.1016/j.foodqual.2012.02.011.

[38] Rose KM, Brossard D, Scheufele DA. Of society, nature, and Health: how perceptions of specific risks and benefits of genetically engineered foods shape public rejection. Environ Commun. 2020;14(7):1017–31. doi: 10.1080/17524032.2019.1710227.

[39] Kato-Nitta N, Tachikawa M, Inagaki Y, Maeda T. Public perceptions of risks and benefits of gene-edited food Crops: an International comparative study between the US, Japan, and Germany. Sci Technol Hum Values. 2022;48(6):1360–92. doi: 10.1177/01622439221123830.

[40] Poortvliet PM, Lokhorst AM. The key role of experiential uncertainty when dealing with risks: its relationships with demand for regulation and institutional trust. Risk Anal. 2016;36(8):1615–29. doi: 10.1111/risa.12543. 26849482

[41] Ahn J, Noh G-Y. Determinants of environmental risk information seeking: an emphasis on institutional trust and personal control. Health Risk Soc. 2020;22(3–4):214–30. doi: 10.1080/13698575.2020.1813261.

[42] Kao Y-H, Sapp SG. The effect of cultural values and institutional trust on public perceptions of government use of network surveillance. Technol Soc. 2022;70:102047. doi: 10.1016/j.techsoc.2022.102047.

[43] Gaskell G, Bauer MW, Durant J, Allum NC. Worlds apart? the reception of genetically modified foods in Europe and the U.S. Science. 1999;285(5426):384–87. doi: 10.1126/science.285.5426.384. 10411496

[44] Dai Y, Huang Y-HC, Jia W, Cai Q. The paradoxical effects of institutional trust on risk perception and risk management in the covid-19 pandemic: evidence from three societies. J Risk Res. 2022;25(11–12):1337–55. doi: 10.1080/13669877.2022.2108122.

[45] Lindberg SA, Peters DJ, Cummings CL. Gene-edited food adoption intentions and institutional trust in the United States: benefits, acceptance, and labeling. Rural Sociol. 2023;88(2):392–425. doi: 10.1111/ruso.12480.

[46] Inna C. Understanding public perception of genetically modified food: navigating misinformation and trust. Food Sci Nutr Technol. 2024;9(3):1–5. doi: 10.23880/fsnt-16000354.

[47] Wu W, Zhang A, van Klinken RD, Schrobback P, Muller JM. Consumer trust in food and the food system: a critical review. Foods. 2021;10(10):2490. doi: 10.3390/foods10102490. 34681539 PMC8536093

[48] Huo L, Liu Y. Genetically modified rice: do Chinese consumers support or go against it? Based on the perspectives of perceived risk and trust. Front Psychol. 2022;13:13. doi: 10.3389/fpsyg.2022.813862. PMC9426681 36051217

[49] Douglas M, Wildavsky A. How can we know the risks we face? Why risk selection is a social Process1. Risk Anal. 1982;2(2):49–58. doi: 10.1111/j.1539-6924.1982.tb01365.x.

[50] Ianis C, Rohmer O, Chauvin B. Cultural values, risk characteristics, and risk perceptions of controversial issues: how does cultural theory work? Risk Anal. 2024;45(3):682–700. doi: 10.1111/risa.17636. 39210832 PMC11954724

[51] David Tàbara J. Precaution and participatory integrated assessment of GM Crops in Spain. Water Sci Technol. 2005;52(6):107–13. doi: 10.2166/wst.2005.0158. 16304942

[52] Gao S, Chen J, Yang Y, Wang G. Understanding the factors driving consumers’ willingness to pay for gene-edited foods in China. Foods. 2024;13(15):2348. doi: 10.3390/foods13152348. 39123540 PMC11311454

[53] Beghin JC, Gustafson CR. Consumer valuation of and attitudes towards novel foods produced with New plant engineering techniques: a review. Sustainability. 2021;13(20):11348. doi: 10.3390/su132011348.

[54] Clark LF. Framing the uncertainty of risk: models of governance for genetically modified foods. Sci Public Policy. 2013;40(4):479–91. doi: 10.1093/scipol/sct001.

[55] Kurzer P, Cooper A. What’s for Dinner?European farming and food traditions confront American biotechnology. Comp Pol Stud. 2007;40(9):1035–58. doi: 10.1177/0010414006288975.

[56] Yang Y, Hobbs JE. How do cultural worldviews shape food technology perceptions? Evidence from a discrete choice experiment. J Agric Econ. 2019;71(2):465–92. doi: 10.1111/1477-9552.12364.

[57] Pellegrini PA. We are all rationalists, but it is not enough: ways of explaining the social acceptance of a theory. Found Sci. 2023;29(4):905–24. doi: 10.1007/s10699-023-09913-0.

[58] Al-Emran M. Beyond technology acceptance: development and evaluation of technology-environmental, economic, and social Sustainability theory. Technol Soc. 2023;75:102383. doi: 10.1016/j.techsoc.2023.102383.

[59] Chan EY, Saqib NU, Gohary A. Social mobility beliefs lower preferences for genetically-modified foods. Appetite. 2025;210:107981. doi: 10.1016/j.appet.2025.107981. 40158700

[60] Lu X, Xie X, Xiong J. Social trust and risk perception of genetically modified food in urban areas of China: the role of salient value similarity. J Risk Res. 2014;18(2):199–214. doi: 10.1080/13669877.2014.889195.

[61] Buddle EA, Fransisca G, Leach J. “They ignore social issues”: understanding the diversity of perspectives on plant gene technologies in Indonesia. Plant Cell Rep. 2025;44(8). doi: 10.1007/s00299-025-03564-0. PMC12283755 40696261

[62] Finucane ML, Holup JL. Psychosocial and cultural factors affecting the perceived risk of genetically modified food: an overview of the literature. Soc Sci Med. 2005;60(7):1603–12. doi: 10.1016/j.socscimed.2004.08.007. 15652691

[63] Cummings C, Peters DJ. Who trusts in gene-edited foods? Analysis of a representative survey study predicting willingness to eat- and purposeful avoidance of gene edited foods in the United States. Front Food Sci Technol. 2022;2. doi: 10.3389/frfst.2022.858277.

[64] Kvakkestad V, Vatn A. Governing uncertain and unknown effects of genetically modified Crops. Ecol Econ. 2011;70(3):524–32. doi: 10.1016/j.ecolecon.2010.10.003.

[65] Kozyreva A, Hertwig R. The interpretation of uncertainty in ecological rationality. Synthese. 2019;198(2):1517–47. doi: 10.1007/s11229-019-02140-w.

[66] Monast JJ, Hemmerich N, Berman ML. Editing nature: reconceptualizing biotechnology governance. Boston Coll Law Rev. 2018;59(7):2377. doi: 10.1056/NEJMp1707409?query=featured_home.

[67] Mandolesi S, Cubero Dudinskaya E, Naspetti S, Solfanelli F, Zanoli R. Freedom of choice—organic consumers’ discourses on New plant breeding techniques. Sustainability. 2022;14(14):8718. doi: 10.3390/su14148718.

[68] Scott SE, Inbar Y, Rozin P. Evidence for absolute moral opposition to genetically modified food in the United States. Perspect Psychol Sci. 2016;11(3):315–24. doi: 10.1177/1745691615621275. 27217243

[69] Thompson PB. Environmental impact and environmental values. Int Lib Environ, Agric Food Ethics/Int Lib Environ, Agric Food Ethics. 2020;32:167–92. doi: 10.1007/978-3-030-61214-6_7.

[70] Durant JR, Evans GA, Thomas GP. The public understanding of science. Nature. 1989;340(6228):11–14. doi: 10.1038/340011a0. 2739718

[71] Wynne B. Misunderstood misunderstanding: social identities and public uptake of science. Public Underst Sci. 1992;1(3):281–304. doi: 10.1088/0963-6625/1/3/004.

[72] Luhmann N, Morgner C, King M. Trust and Power. Cambridge: Polity Press; 2018.

[73] Cugueró N, Rosanas JM. Trust under bounded rationality: competence, value systems, unselfishness and the development of virtue. Intangible Capital. 2019;15(1):1. doi: 10.3926/ic.1407.

[74] Xu X, Zhang Q, Chen X. Consensus-based non-cooperative behaviors management in large-group emergency decision-making considering experts’ trust relations and preference risks. Knowl-Based Syst. 2020;190:105108. doi: 10.1016/j.knosys.2019.105108.

[75] Peng L, Tan J, Lin L, Xu D. Understanding sustainable disaster mitigation of stakeholder engagement: risk perception, trust in public institutions, and disaster insurance. Sustain Dev. 2019;27(5):885–97. doi: 10.1002/sd.1948.

[76] Schroeder SA. Democratic values: a better foundation for public trust in science. Br J Philos Sci. 2021;72(2):545–62. doi: 10.1093/bjps/axz023.

[77] Brom FWA. Food, Consumer concerns, and trust: food ethics for a globalizing market. J Agric Environ Ethics. 2000;12(2):127–39. doi: 10.1023/a:1009586529518.

[78] Jensen KK, Sandøe P. Food safety and ethics: the interplay between science and values. J Agric Environ Ethics. 2002;15(3):245–53. doi: 10.1023/a:1015726423707.

[79] Lazaroiu G, Andronie M, Uţă C, Hurloiu I. Trust management in organic agriculture: sustainable consumption Behavior, environmentally conscious purchase intention, and healthy food choices. Front Public Health. 2019;7:7. doi: 10.3389/fpubh.2019.00340. 31803705 PMC6877712

[80] Bredahl L. Determinants of Consumer attitudes and purchase intentions with regard to genetically modified food – results of a cross-national survey. J Consum Policy (Dordr). 2001;24(1):23–61. doi: 10.1023/a:1010950406128.

[81] Siegrist M. The influence of trust and perceptions of risks and benefits on the acceptance of gene technology. Risk Anal. 2000;20(2):195–204. doi: 10.1111/0272-4332.202020. 10859780

[82] Pang Z, Ye Z. The government trust structure dimension behind public attitude to genetically modified agricultural technology: a qualitative study based on Weibo comments(In Chinese). Forum Sci Technol China. 2019;8:114–22. doi: 10.13580/j.cnki.fstc.2019.08.015.

[83] Todd M, Fiddick L, Krauss S. Ecological rationality and its contents. Thinking Reasoning. 2000;6(4):375–84. doi: 10.1080/135467800750038184.

[84] Slovic P, Finucane ML, Peters E, MacGregor DG. Risk as analysis and risk as feelings: some thoughts about affect, reason, risk, and rationality. Risk Anal. 2004;24(2):311–22. doi: 10.1111/j.0272-4332.2004.00433.x. 15078302

[85] Bearth A, Siegrist M. Are risk or benefit perceptions more important for public acceptance of innovative food technologies: a meta-analysis. Trends Food Sci Technol. 2016;49(49):14–23. doi: 10.1016/j.tifs.2016.01.003.

[86] Morelli M, Casagrande M, Forte G. Decision making: a theoretical review. Integr Psychol Behav Sci. 2021;56(3):609–29. doi: 10.1007/s12124-021-09669-x. 34780011

[87] Wang S. Hostile media perceptions and consumption of genetically modified and organic foods: examining the mediating role of risk-benefit assessments. Risk Anal. 2022;43(8):1587–98. doi: 10.1111/risa.14054. 36307377

[88] Jia H. Science in movements: knowledge control and social contestation in China’s hydropower, GMO and nuclear controversies. London: Routledge; 2021.

[89] Kasperson RE, Webler T, Ram B, Sutton J. The social amplification of risk framework: new perspectives. Risk Anal. 2022;42(7):1367–80. doi: 10.1111/risa.13926. 35861634 PMC10360138

[90] Hwang H, Nam S-J. The influence of consumers’ knowledge on their responses to genetically modified foods. GM Crops Food. 2020;12(1):146–57. doi: 10.1080/21645698.2020.1840911. PMC7644159 33138666

[91] Seidl R, Drögemüller C. Procedural fairness and safety in the acceptance of nuclear waste disposal in Germany: an empirical study. J Risk Res. 2024;27(8):969–85. doi: 10.1080/13669877.2024.2431896.

[92] Werth JC. Negotiating the ethics of GM foods. BRILL EBooks. 2022;77:252–73. doi: 10.1163/9789004524705_014.

[93] Green NL. An analysis of some ethical argumentation about genetically modified food. Argument Computation. 2023;15(1):1–20. doi: 10.3233/aac-220014.

[94] Zhaleh R, Mohammadi H, Boccia F, Firoozzare A, Covino D. Consumption of genetically modified food products and its Determinants (case study: edible oil in Mashhad). Foods. 2023;12(15):2933. doi: 10.3390/foods12152933. 37569202 PMC10417801

[95] Siegrist M. Trust and risk perception: a critical review of the literature. Risk Anal. 2019;41(3):480–90. doi: 10.1111/risa.13325. 31046144

[96] Hakim MP, Zanetta LD, de Oliveira JM, da Cunha DT. The mandatory labeling of genetically modified foods in Brazil: Consumer’s knowledge, trust, and risk perception. Food Res Int. 2020;132:109053. doi: 10.1016/j.foodres.2020.109053. 32331628

[97] Ali S, Nawaz MA, Ghufran M, Hussain SN, Hussein Mohammed AS. GM trust shaped by trust Determinants with the impact of risk/benefit framework: the contingent role of food technology neophobia. GM Crops Food. 2020;12(1):170–91. doi: 10.1080/21645698.2020.1848230. PMC7781675 33356819

[98] Mohd L, Tan Y S. & Chai LC. Unveiling food safety perceptions: a study on Consumer Behavior and trust in Malaysia. Qual Assur Saf Crops Foods. 2025;17(3):359–74. doi: 10.15586/qas.v17i3.1569.

[99] Ari E, Yilmaz V, Olgun M. The effect of trust benefit and risk perception of GM foods on Behavior intention: a study on University students. J Economy Culture Soc. 2021;(64):297–312. doi: 10.26650/jecs2021-930755.

